# Supplementary material for: Iterative improvement in the automatic modular design of robot swarms
Source: PeerJ Comput Sci. 2020 Dec 7;6:e322. doi: 10.7717/peerj-cs.322 (PMC7924708; doi:10.7717/peerj-cs.322)
Supplement: Supplemental Information 3 [file peerj-cs-06-322-s003.zip › argos3/doc/api/standalone/a00554_source.html]

ARGoS: plugins/robots/mini-quadrotor/simulator/physx\_miniquadrotor\_model.cpp Source File


- Main Page
- Related Pages
- Namespaces
- Classes
- Files

- File List
- File Members

# plugins/robots/mini-quadrotor/simulator/physx\_miniquadrotor\_model.cpp

Go to the documentation of this file.

```
00001 
00007 #include "physx_miniquadrotor_model.h"
00008 #include <argos3/plugins/robots/mini-quadrotor/simulator/miniquadrotor_entity.h>
00009 #include <argos3/plugins/simulator/entities/rotor_equipped_entity.h>
00010 
00011 namespace argos {
00012 
00013    /****************************************/
00014    /****************************************/
00015    
00016    /*
00017     * The parameter values are taken from data provided by Kumar's group directly
00018     */
00019    static const Real ARM_LENGTH            = 0.063f;
00020    static const Real ARM_HALF_LENGTH       = ARM_LENGTH * 0.5f;
00021    static const Real PROPELLER_LENGTH      = 0.082f;
00022    static const Real PROPELLER_HALF_LENGTH = PROPELLER_LENGTH * 0.5f;
00023    static const Real BODY_HALF_WIDTH       = ARM_HALF_LENGTH + PROPELLER_HALF_LENGTH;
00024    static const Real BODY_HEIGHT           = 0.015f;
00025    static const Real BODY_HALF_HEIGHT      = BODY_HEIGHT * 0.5f;
00026    static const Real BODY_MASS             = 0.06736f;
00027    static const Real UPLIFT_COEFFICIENT    = 1.9985e-9;
00028    static const Real DRAG_COEFFICIENT      = 4.737e-12;
00029 
00030    static const physx::PxVec3 POSITION_ERROR_COEFF(6.61f, 6.61f, 72.0f); // unused
00031    static const physx::PxVec3 VELOCITY_ERROR_COEFF(5.14f, 5.14f, 24.0f); // unused
00032 
00033    static const physx::PxTransform PITCH_ARM_POSE(physx::PxQuat(physx::PxHalfPi, physx::PxVec3(0.0f, 0.0f, 1.0f)));
00034    static const physx::PxVec3      INERTIA_TENSOR_DIAGONAL(2.32e-3, 2.32e-3, 4e-3);
00035    static const physx::PxMat33     INERTIA_TENSOR(physx::PxMat33::createDiagonal(INERTIA_TENSOR_DIAGONAL));
00036    static const physx::PxMat33     INERTIA_TENSOR_INVERSE(INERTIA_TENSOR.getInverse());
00037 
00038    /****************************************/
00039    /****************************************/
00040 
00041    CPhysXMiniQuadrotorModel::CPhysXMiniQuadrotorModel(CPhysXEngine& c_engine,
00042                                                       CMiniQuadrotorEntity& c_entity) :
00043       CPhysXSingleBodyObjectModel(c_engine, c_entity),
00044       m_cMiniQuadrotorEntity(c_entity) {
00045       /* Calculate base center */
00046       SetARGoSReferencePoint(physx::PxVec3(0.0f, 0.0f, -BODY_HALF_HEIGHT));
00047       /* Get position and orientation in this engine's representation */
00048       physx::PxVec3 cPos;
00049       CVector3ToPxVec3(GetEmbodiedEntity().GetOriginAnchor().Position, cPos);
00050       physx::PxQuat cOrient;
00051       CQuaternionToPxQuat(GetEmbodiedEntity().GetOriginAnchor().Orientation, cOrient);
00052       /* Create the transform
00053        * 1. a translation up by half body height
00054        * 2. a rotation around the base
00055        * 3. a translation to the final position
00056        */
00057       physx::PxTransform cTranslation1(physx::PxVec3(0.0f, 0.0f, BODY_HALF_HEIGHT));
00058       physx::PxTransform cRotation(cOrient);
00059       physx::PxTransform cTranslation2(cPos);
00060       physx::PxTransform cFinalTrans = cTranslation2 * cRotation * cTranslation1;
00061       /* Create the capsule geometry for the arms */
00062       physx::PxCapsuleGeometry cArmGeometry(BODY_HALF_HEIGHT,
00063                                             BODY_HALF_WIDTH);
00064       /* Create the body in its initial position and orientation */
00065       m_pcBody = GetPhysXEngine().GetPhysics().createRigidDynamic(cFinalTrans);
00066       /* Enable CCD on the body */
00067       m_pcBody->setRigidBodyFlag(physx::PxRigidBodyFlag::eENABLE_CCD, true);
00068       /* Create the shape for the roll arm */
00069       physx::PxShape* pcRollArmShape =
00070          m_pcBody->createShape(cArmGeometry,
00071                                GetPhysXEngine().GetDefaultMaterial());
00072       pcRollArmShape->userData = this;
00073       /* Create the shape for the pitch arm */
00074       physx::PxShape* pcPitchArmShape =
00075          m_pcBody->createShape(cArmGeometry,
00076                                GetPhysXEngine().GetDefaultMaterial(),
00077                                PITCH_ARM_POSE);
00078       pcPitchArmShape->userData = this;
00079       /* Set body mass and inertia tensor */
00080       m_pcBody->setMass(BODY_MASS);
00081       m_pcBody->setMassSpaceInertiaTensor(INERTIA_TENSOR_DIAGONAL);
00082       /* Add body to the scene */
00083       GetPhysXEngine().GetScene().addActor(*m_pcBody);
00084       /* Setup the body model data */
00085       SetupBody(m_pcBody);
00086       /* Now we can calculate the bounding box */
00087       CalculateBoundingBox();
00088    }
00089 
00090    /****************************************/
00091    /****************************************/
00092 
00093    void CPhysXMiniQuadrotorModel::UpdateFromEntityStatus() {
00094       /* Get desired rotor velocities */
00095       const Real* pfRotorVel =
00096          m_cMiniQuadrotorEntity.GetRotorEquippedEntity().GetRotorVelocities();
00097       /* Calculate the squares */
00098       Real pfSquareRotorVel[4] = {
00099          pfRotorVel[0] * pfRotorVel[0],
00100          pfRotorVel[1] * pfRotorVel[1],
00101          pfRotorVel[2] * pfRotorVel[2],
00102          pfRotorVel[3] * pfRotorVel[3]
00103       };
00104       /* Calculate uplift-related control input */
00105       Real fUpliftInput =
00106          UPLIFT_COEFFICIENT *
00107          ((pfSquareRotorVel[0]) +
00108           (pfSquareRotorVel[1]) +
00109           (pfSquareRotorVel[2]) +
00110           (pfSquareRotorVel[3]));
00111       /* Calculate torque-related control inputs */
00112       physx::PxVec3 cTorqueInputs(
00113          UPLIFT_COEFFICIENT * ARM_HALF_LENGTH * (pfSquareRotorVel[0] - pfSquareRotorVel[2]),
00114          UPLIFT_COEFFICIENT * ARM_HALF_LENGTH * (pfSquareRotorVel[1] - pfSquareRotorVel[3]),
00115          DRAG_COEFFICIENT * (pfSquareRotorVel[0] - pfSquareRotorVel[1] + pfSquareRotorVel[2] - pfSquareRotorVel[3]));
00116       /* Apply uplift */
00117       physx::PxRigidBodyExt::addLocalForceAtLocalPos(
00118          *m_pcBody,
00119          physx::PxVec3(0.0f, 0.0f, fUpliftInput),
00120          physx::PxVec3(0.0f));
00121       /* Apply rotational moment */
00122       physx::PxVec3 cTorque = (-m_pcBody->getAngularVelocity()).cross(INERTIA_TENSOR * m_pcBody->getAngularVelocity()) + cTorqueInputs;
00123       m_pcBody->addTorque(cTorque);
00124    }
00125 
00126    /****************************************/
00127    /****************************************/
00128 
00129    REGISTER_STANDARD_PHYSX_OPERATIONS_ON_ENTITY(CMiniQuadrotorEntity, CPhysXMiniQuadrotorModel);
00130 
00131    /****************************************/
00132    /****************************************/
00133 
00134 }
```

---

Generated on 10 Jul 2018 for ARGoS by 
 1.6.1
